# An Extended Passive Motion Paradigm for Human-Like Posture and Movement Planning in Redundant Manipulators

**DOI:** 10.3389/fnbot.2017.00065

**Published:** 2017-11-30

**Authors:** Paolo Tommasino, Domenico Campolo

**Affiliations:** ^1^Laboratory of Neuromotor Physiology, Fondazione Santa Lucia, Rome, Italy; ^2^Synergy Lab, Robotics Research Centre, School of Mechanical and Aerospace Engineering, Nanyang Technological University, Singapore, Singapore

**Keywords:** kinematic redundancy, postural synergies, Donders' law, posture, movement, pointing

## Abstract

A major challenge in robotics and computational neuroscience is relative to the posture/movement problem in presence of kinematic redundancy. We recently addressed this issue using a principled approach which, in conjunction with nonlinear inverse optimization, allowed capturing postural strategies such as Donders' law. In this work, after presenting this general model specifying it as an extension of the Passive Motion Paradigm, we show how, once fitted to capture experimental postural strategies, the model is actually able to also predict movements. More specifically, the passive motion paradigm embeds two main intrinsic components: joint damping and joint stiffness. In previous work we showed that joint stiffness is responsible for static postures and, in this sense, its parameters are regressed to fit to experimental postural strategies. Here, we show how joint damping, in particular its anisotropy, directly affects task-space movements. Rather than using damping parameters to fit *a posteriori* task-space motions, we make the *a priori* hypothesis that damping is proportional to stiffness. This remarkably allows a postural-fitted model to also capture dynamic performance such as curvature and hysteresis of task-space trajectories during wrist pointing tasks, confirming and extending previous findings in literature.

## 1. Introduction

Recent trends in both industry and healthcare clearly show the need for robots to be able to cooperate and assist humans in specific tasks. In order to do so, not only our robots will need to be safe-by-design, incorporating for example compliant mechanisms (Haddadin et al., 2010; Vanderborght et al., 2013) and force/impedance control architectures (Ficuciello et al., 2015) (as opposed to the current rigid and position-controlled deployed in industry) but will also need to *behave* naturally. In other words, while working with a robot, human operators not only need to be safe at all times, but shall also feel comfortable. As an example, imagine a robotic assistant designed to hand-over tools to a human operator. It is quite important for the robot to assume *natural postures*, which carry non-verbal semantics very valuable to human operators (the same object can be passed in different ways, for different purposes). For this and other reasons, in the last decades, roboticists have started looking into human motor strategies as a source of inspiration for the formulation of bio-inspired postural/motion controllers (Khatib et al., 2004; Schaal and Schweighofer, 2005; Kim et al., 2011, 2013; Zanchettin et al., 2013).

Another characteristic of modern robots is that they are general-purpose (unlike, for example, a CNC machine) and often bear human-like functionalities, if not resemblance. In this paper we shall be mainly interested in robotic manipulators. Currently, many commercial robotic arms are made available (in single or bimanual configuration) with kinematic similarities to human arms (Smith and Rooks, 2006; Albu-Schaffer et al., 2008). One of the specific similarities lies in the degrees-of-freedom (DOF), typically 6–7 in current robotic manipulators, and the *kinematic redundancy* which comes with it when dealing with most of tasks. The coordination of redundant degrees-of-freedom is a central topic in both robotics and neuroscience and we are interested in two specific aspects: the *redundancy problem* (Bernstein, 1967) and the *posture/movement problem* (Ostry and Feldman, 2003). This issue was first addressed by the authors in a recent work (Tommasino and Campolo, 2017) where a principled approach was proposed to tackle these very issues with focus on capturing human-like postural strategies: static (or equilibrium) postures satisfying a given (static) task constraint. In this work, we shall specialize the computational model and extend our previous results to the problem of movement generation: given a desired task constraint, find human-like motions (and postures), both in task and joint space, that brings the current robot posture to the desired task-space target.

### The robotics approach to kinematic redundancy

Motions for robotic manipulators are typically planned in task-space as it is much easier and intuitive to define a trajectory for a robotic end-effector than for its multiple (and often) redundant joints. For example, if we want a robotic manipulator to reach for a given object we can easily program the robotic gripper to follow a desired task-space path ***x***_*d*_ with a given task-space velocity ***ẋ***_*d*_ rather than programming the trajectory of each individual joint. However, due to kinematic redundancy, mapping the desired task trajectory in joint space is challenging as infinite combinations of joints trajectories are possible for the same task-space trajectory. This issue has a very long history in robotics and it has been tackled by roboticists, either at the kinematic or at the force level, with a local optimization approach and the use of weighted pseudo-inverses of the Jacobian matrix of the robot manipulator (Klein and Huang, 1983; Nenchev, 1989; English and Maciejewski, 2000). For instance, a simple way to map a desired task trajectory ***ẋ***_*d*_ in joint space is: ${\dot{q}}_{d}=J_{W}^{\#}\left(\text{q}\right){ẋ}_{d}$, where $J_{W}^{\#}$ is any W-weighted generalized pseudo-inverse of the Jacobian matrix *J*. However, it was soon realized that such solution, although simple, very often results in non-holonomic, or non-repeatable joint trajectories, i.e., the robot equilibrium posture satisfying a given task-constraint is not unique but depends on the path that robot followed before reaching the desired task constraint (Klein and Huang, 1983; Mussa-Ivaldi and Hogan, 1991). This type of solution is problematic especially for cyclic task-space movements as non-repeatable joint motions can result in instability and/or violations of joint constraints. At the kinematic level, the problem of repeatability can be tackled by planning an additional joint trajectory (or null-space motion) ${\dot{q}}_{0}$ that does not interfere with the planned task-space motions **ẋ**: $\dot{q}=J_{W}^{\#}\left(\text{q}\right)ẋ+N_{W}\left(\text{q}\right){\dot{q}}_{0}$ where *N*_*W*_(***q***) is the null-space projector operator associated to the weighting matrix *W* (Klein and Huang, 1983; Nenchev, 1989; English and Maciejewski, 2000)^1^. While kinematic motion planning requires an *execution* level to track the desired trajectory in joint space (such as computed torque control or PD control (Murray et al., 1994), weighted pseudo-inverses and null-space projectors can also be used to solve kinematic redundancy at the force/torque level: $\tau =J^{T}\left(\text{q}\right)\text{F}+N_{W}^{T}\nabla_{\text{q}}h\left(\text{q}\right)$, where **τ** is the commanded joint torque, ***F*** is a task-space force fields (Mistry and Schaal, 2015)^2^ that drives the robotic end-effector along desired task constraints and ∇_***q***_*h*(***q***) is the gradient of a real or virtual potential fields that is mapped in the null-space of the Jacobian transpose matrix to achieve repeatable joint motions.

In the last decade, task-space control has been extensively used in robotics to generate human-like and/or adaptive robot behavior (Schaal and Schweighofer, 2005) either at the tasks-space level, in terms of adaptive trajectories (Peters and Schaal, 2007; Degallier and Ijspeert, 2010) and Cartesian impedance (Calinon et al., 2013), then at the joint space level in terms of null-space control and weighting matrix *W* (Khatib et al., 2004; Nakanishi et al., 2008; Dietrich et al., 2015).

### Kinematic constraints and computational approaches to human motor control

In neuroscience is still debated whether the human brain adopts a hierarchical approach to plan and control movements and whether the brain plans and control task-space and null-space motions independently (Jordan and Wolpert, 1999; Mussa-Ivaldi et al., 2011; Mistry and Schaal, 2015). The experimental evidence that unconstrained planar reaching movements features straight-line paths and bell-shaped velocity profiles led to the hypothesis that the human brain plans hand movement in task-space, by shifting the equilibrium position of the hand according to a minimum-jerk trajectory. This trajectory would then be tracked in joint space (hence at a lower level) by an impedance controller that exploits muscle visco-elasticity [see the Equilibrium-Point Hypothesis (EPH); Flash, 1987 for more details]. Later studies however, showed that in other experimental conditions hand movements were curved and models such as the minimum-torque change (Uno et al., 1989) and the minimum-variance (Harris and Wolpert, 1998) were able to capture these human movement features by solving an optimal control problem directly in joint space.

#### Postural synergies: Donders' law, uncontrolled manifold and the leading joint hypothesis

Postures are somewhat *static*, possibly accounted for as equilibria of some potential field (Campolo et al., 2011), while movement is in apparent contrast with the very concept of equilibrium. The Posture/Movement problem stems out from the possible interference of postural control mechanisms with general motor strategies (Ostry and Feldman, 2003). In the last few decades, various approaches have been proposed in computational neuroscience as an attempt to reconcile posture and movement.

An extensive number of behavioral studies have shown that, at the joint-space level, during kinematically redundant tasks, humans adopt a stereotypical strategy that associates a *unique* and *path-independent* posture to a given task (Hepp, 1990; Haslwanter, 1995). This kinematic strategy is usually called *Donders' law*, since the Dutch ophthalmologist Donders showed (1847) that for any steady gazing direction (task), the human eye assumes a unique combination of elevation, azimuth, and torsion angles (posture). Donders-like strategies have also been found for pointing tasks involving the head (Ceylan et al., 2000; Crawford et al., 2003), the wrist (Campolo et al., 2009), the shoulder (Hore et al., 1992) and for pointing/reaching tasks involving the upper arm (Liebermann et al., 2006; Ewart et al., 2016).

It has been suggested that the brain implements Donders' Law as a flexible family of *holonomic constraints* (Medendorp et al., 2000; Crawford et al., 2003) to solve redundancy as well as to fulfill some *optimality* criteria that might vary in different experimental scenarios and physiological conditions (Ceylan et al., 2000; Medendorp et al., 2000; Wong, 2004).

From a computational perspective, Donders-like postural strategies, can be captured by solving a constrained optimization problem which returns the unique optimal posture that minimizes a given (posture-dependent) objective function while fulfilling a desired task-constraint (Cruse et al., 1990; De Sapio et al., 2006; Campolo et al., 2011). Because this type of *postural models* only computes static/equilibrium-configurations they are usually not suitable for planning movements. *Transport models* (Vetter et al., 2002) such as minimum-torque-change (Uno et al., 1989), minimum-work (Soechting et al., 1995), minimum-variance (Vetter et al., 2002), do provide a solution to the Posture/Movement problem but, in their original formulation are *incompatible* with Donders' law as they predict path-dependent equilibrium postures (Admiraal et al., 2004).

Kinematic constraints such as Donders' law, suggest that the brain may plan and control equilibrium postures directly in joint-space, by constraining redundant postures to a sub-manifold (Donders' surface) of the joint-space. Experimental studies involving redundant DOFs however, have also shown that motor variability is always higher along *task-irrelevant* directions (also known in human motor control as *uncontrolled manifold*) of the joint-space rather than along task-relevant directions (Latash et al., 2007). These results led to the Uncontrolled Manifold hypothesis (Scholz and Schöner, 1999) according to which the brain does not freeze redundant DOFs into a holonomic constraint (such as Donders' law) but instead uses redundant DOFs to push “bad motor variability” (i.e., directly affecting the task) along task-irrelevant directions of the joint space. In other words, according to the UCM hypothesis the brain would only stabilize elemental variables (such as joint rotations) that directly affect task performance while leaving task-irrelevant directions *uncontrolled*.

An alternative theory on how the brain may simplify the control of redundant DOFs is the Leading Joint Hypothesis (LJH) (Dounskaia, 2005). Central to the theory is the fact that link segments are coupled to each other by non-linear interaction torques so that motion in one joint unavoidably introduce motions to nearby joints, especially for fast speed movements. According to the LJH, the brain, depending on the specific task, organizes joints hierarchically: the “leading” joint, typically a proximal joint of the chain, is accelerated/decelerated as in a single joint movement, hence neglecting interaction torques and motions at the other joints. Subordinate joints instead, “monitor the interaction torque effect and create net torque that results in limb motion characteristics required by the task, including movement direction, accuracy, and so on.” Although in line with intuition, the LJH, to the best of authors knowledge, does not really propose a computational framework.

#### Optimal feedback control and passive motion paradigm

The UCM and the LJH do provide theories of human motor control and mathematical frameworks to analyse human movements in terms of joint variability and leading/subordinate joints respectively. However, very little is known on how the brain may actually implement such motor strategies. Optimal feedback control (OFC) is probably one of the most accredited computational model of human motor control that can reproduce both average trajectories of human reaching movements and, to some extend, can predict the patterns of motor variability typical of the UCM hypothesis (Todorov and Jordan, 2002). Contrary to the robotics task-space control, in the OFC framework there is no distinction between planning and execution and task and joint space trajectories simply unfolds as the optimal controller adjusts feedback gains to suit the overall goals of the system. The OFC also predicts movement variability in line with the UCM hypothesis as deviations from the average trajectory are not correct by the controller if they do not affect task performance (minimum intervention principle).

Although the OFC framework has been very successful at modeling movement strategies typical of planar non-redundant point-to-point reaching movements (Scott, 2004), some computational studies have reported difficulties in solving optimal control problem in the presence of both kinematic redundancy and static forces (gravitational and/or elastic). This is because, to hold the body still at equilibrium (i.e., at the end of a movement), suitable boundary conditions must be specified so that the optimal muscle forces can counterbalance the static forces acting on the body (see Guigon et al., 2007 and references therein). Recently, this Posture/Movement problem has been tackled with the Separation Principle according to which the brain processes static (i.e., configuration-dependent) and dynamic (i.e., velocity-dependent) joint torques separately (Hollerbach and Flash, 1982; Atkeson and Hollerbach, 1985; Guigon et al., 2007). By combining the optimal control framework with the Separation Principle, Guigon and colleagues were able to implement human-like motor strategies in redundant manipulators (Guigon et al., 2007; Taïx et al., 2013). However, their approach, formulated at the joint-space level, results into path-dependent (i.e., dependent on the movement history and initial body configuration) terminal postures and therefore such an approach cannot predict kinematic synergies such as Donders' law.

An alternative theory to the OFC framework is the so-called Passive Motion Paradigm (PMP) (Mussa-Ivaldi et al., 1988; Mohan and Morasso, 2011; Morasso et al., 2015) PMP can be considered a computational generalization of the EPH, in that *goals and kinematic constraints can be superimposed when viewed as force-fields*. First proposed in the 80 s (Mussa-Ivaldi et al., 1988), the PMP has evolved over the years and has been proposed as a theory of human trajectory formation and as bio-inspired trajectory planner for redundant robots (Mohan and Morasso, 2007; Morasso et al., 2010; Mohan et al., 2011).

**Figure 1:**
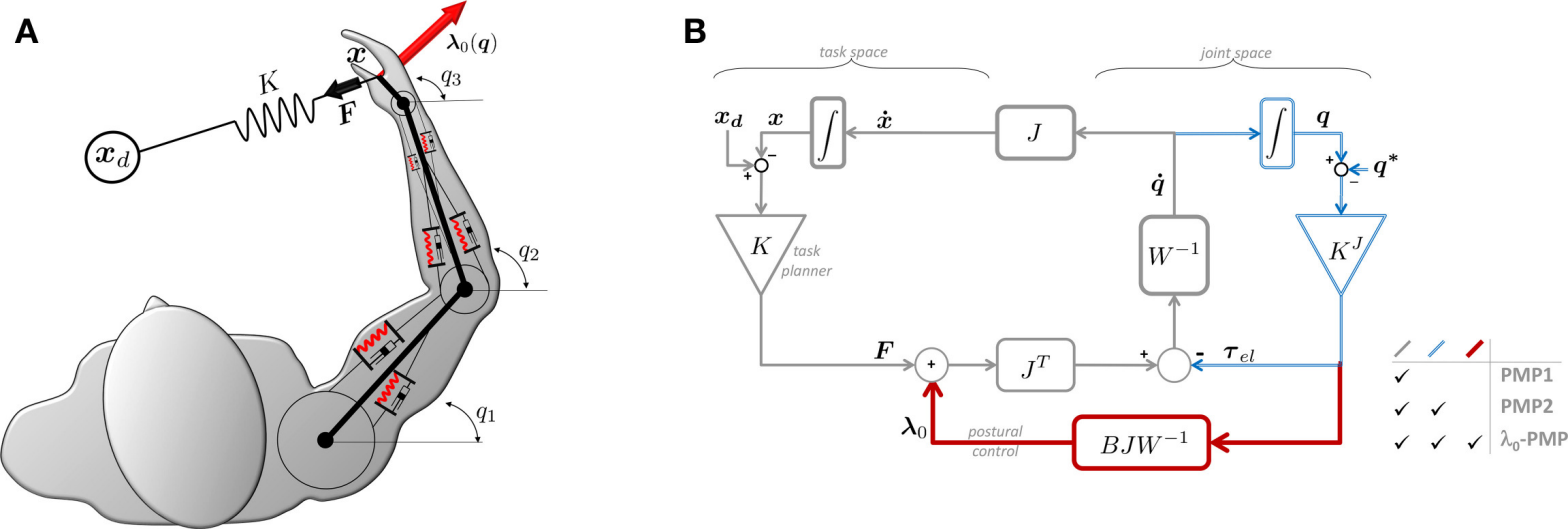
**λ**_0_-PMP as an extension of the Passive Motion Paradigm. **(A)** Example of a 3DOF human-like arm performing a redundant 2D reaching task. **(B)** Block diagram of the general **λ**_0_-PMP model.

One of the major strengths of the PMP lies in its computational simplicity. While full details of the PMP are found in Mohan et al. (2011) and references therein, its basic features are illustrated in Figure 1. In the standard PMP model, a robotic manipulator is seen as a rigid structure (i.e., an arm-like kinematic chain) with “intrinsic” properties defined at the level of joint-space (e.g., joint angles *q*_1_, *q*_2_, *q*_3_) and “extrinsic” properties defined at the level of task-space (e.g., actual and desired endpoint postures ***x*** and ***x***_*d*_, respectively). The redundancy problem is solved by postural mechanisms implemented via the action of an *intrinsic impedance*, for example in the form of purely viscous (mechanical dampers) or viscoelastic (dampers and springs) elements interconnected at joint level. On the other hand, movement is planned at task-level and implemented by an *extrinsic impedance*, ***F*** = *K*(***x***_*d*_ − ***x***) in Figure 1, acting as a generalized spring which continuously drives the end-effector (at some position ***x***) toward the goal ***x***_*d*_ while the intrinsic impedance takes care of postures.

The standard PMP comes in two forms, with the only difference in terms of intrinsic impedance: one being *purely viscous* and the other being *viscoelastic*. In the first case, it can be easily shown (Tommasino and Campolo, 2017) that a purely viscous intrinsic impedance solves the posture/movement problem but is *incompatible* with Donders' law (as it does not yield repeatable postures). In the second case, the intrinsic viscoelastic impedance ensures unique postures (due to an elastic potential in joint-space) but does not solve the posture/movement problem. A simple way to see this is that the intrinsic springs “pull” the end-effector back to a rest position (q^*^), in contrast with the extrinsic spring which pulls the end-effector toward a target ***x***_*d*_. To ensure task completion (i.e., ***x*** = ***x***_*d*_) one should set the target at a different location, say ***x***_*d*′_, so that the end-effector ends up being in equilibrium at the planned target ***x***_*d*_. Computing ***x***_*d*′_ is not trivial and somewhat blurs the separation between task and posture as ***x***_*d*′_ depends on both the intrinsic and the extrinsic elastic potentials.

### An extended passive motion paradigm (λ_0_-PMP)

One way to prevent the interference between intrinsic and extrinsic elastic potentials is to *block any effect of the intrinsic potential onto the task*. Inspired by the *Separation Principle* (of static and dynamic torques) (Guigon et al., 2007), we recently proposed the **λ**_0_-PMP model (Tommasino and Campolo, 2017), an extension of the standard PMP. Experimental evidence shows that the human brain processes *static* (or configuration-dependent) and *dynamic* (or velocity-dependent) *force fields separately* (Hollerbach and Flash, 1982; Atkeson and Hollerbach, 1985; Nishikawa et al., 1999; Kurtzer et al., 2005b). Because static forces such as gravitational or elastic fields are predominant during slow movements and are not affected by movement speed, the Separation Principle (Guigon et al., 2007) has been proposed as a simplifying control strategy for the brain to learn new movements (Nishikawa et al., 1999), to efficiently time-scale arm trajectories (Hollerbach and Flash, 1982; Atkeson and Hollerbach, 1985) and to robustly cope with the effect of gravity in different environments (Kurtzer et al., 2005a).

In literature, the Separation Principle is typically applied at joint-space level (larger than task-space, dimension-wise, when dealing with redundant manipulators), assuming that static contributions (either due to gravity or to elastic fields) are perfectly compensated for by the brain (or by the robot controller) so that they can be removed from the dynamic equations of the limb under control (Guigon et al., 2007; Taïx et al., 2013). In our recent work (Tommasino and Campolo, 2017), we derived the **λ**_0_-PMP model by (i) re-framing the problem within the *constrained minimization framework* and applied the Lagrange Multipliers method; (ii) noting that the Lagrange Multipliers **λ** define a *task-space force field*; (iii) applying the Separation Principle to **λ** and defining a *static task-space force field* **λ**_0_ (from which the name of the method). This task-space force **λ**_0_, also highlighted in Figure 1 as an addition to the standard PMP, produces *partial compensation* of joint torques, blocking their effect *only* on the tasks space, leaving joint torques free to act in the *null-space*, and driving the posture toward minima of the potential without interfering with task-space objectives. This captures the essence of postural synergies such as Donders' law which can now be seen as generated from a joint-space potential combined with a task-space force field.

### Scope of this work and contribution

With reference to Figure 1B, for the **λ**_0_-PMP, once the Jacobian matrix *J* is defined (given the geometry of the manipulator and of the task), the parameters which need to be determined are the *intrinsic stiffness matrix K*^*J*^; the *intrinsic damping matrix W* (or, equivalently, the admittance *W*^−1^); and the *extrinsic stiffness matrix K*. Although all these intrinsic and extrinsic parameters are required to plan motion, this work will focus on the role of intrinsic properties and in particular the intrinsic damping matrix *W*.

In previous works, we focused on postural strategies and showed how Donders' Law can be captured via an intrinsic elastic potential (Campolo et al., 2011; Tommasino and Campolo, 2016) and how nonlinear inverse optimization can be used to determine the coefficients of the intrinsic stiffness *K*^*J*^ to fit experimental data (Tommasino and Campolo, 2017). In this work, we shift our focus on movement dynamics, which are primarily shaped by the damping matrix *W*. Rather than trying to use the coefficients of the matrix *W* as “extra degrees of freedom” to better fit experimental data *a posteriori*, we assume *a priori* that *damping is proportional to stiffness*, in line with experimental evidence (Tsuji et al., 1995; Perreault et al., 2004; Tee et al., 2004; Peaden and Charles, 2014). In other words, we hypothesize that the same biomechanical factors which determine the “shape” of *K*^*J*^ (i.e., its eigenvectors and eigenvalues) also determine the “shape” of *W*.

With this hypothesis in place, the intrinsic stiffness still has an “indirect effect” as it shapes the intrinsic damping matrix *W*, whose dynamic effects are not blocked by **λ**_0_. We shall specifically show how this mechanism determines curvature of task-space trajectories during pointing tasks performed with the wrist, in line with the experimental evidence also reported in literature by Charles and Hogan (2010). Lastly, it should be noted that any stable task-space force field can be used as a movement planner and some possible choices have already been reported in Tommasino and Campolo (2017). In line with the PMP, in this work we assume that the task planner is a *virtual* elastic field driving the end-effector toward the desired target. A detailed comparison of different task-space planners will be reported in a separate work.

## 2. Materials and methods

This section presents the **λ**_0_-PMP, a novel extension of the Passive Motion Paradigm, and its specialization to wrist pointing tasks which will be later used in a comparative analysis with experimental data.

Although bearing remarkable similarities with the Passive Motion Paradigm, the theoretical derivation of the **λ**_0_-PMP follows a principled approach, described in detail in Tommasino and Campolo (2017). However, this similarity allows presenting our model as an extension of the PMP, facilitating readers already familiar with the Passive Motion Paradigm itself. To this end, Figure 1B highlights the differences between the two standard PMP models (here denoted as PMP1 and PMP2) and ours.

### λ_0_-PMP

One of the computational advantages of the PMP is its *ability to solve the redundancy problem* without explicit kinematic inversion and cost function computation (Mohan and Morasso, 2011). To see how this is accomplished, consider a redundant manipulator with forward kinematics

(1) $$\begin{array}{l} \text{x}=FK\left(\text{q}\right) \end{array}$$

where ***x*** ∈ ℝ^*m*^ is a given end-effector pose, ***q*** ∈ ℝ^*n*^ is a given manipulator configuration and the inequality *m* < *n* denotes kinematic redundancy. As an example, Figure 1A shows a planar human-like arm with a three-dimensional joint space (consisting of three rotational joints $\text{q}={\left[q_{1}\;q_{2}\;q_{3}\right]}^{T}$) and with a two-dimensional task-space encoding of the actual hand position ***x*** and the desired hand position ***x***_*d*_. Once the Forward Kinematics (*FK*) of the manipulator is defined in relation to a specific task, one can compute the *task Jacobian*, an *n* × *m* matrix which maps joint space velocities $\dot{q}$ into task-space velocities **ẋ**:

(2) $$\begin{array}{l} ẋ=\frac{\partial FK}{\partial \text{q}}\dot{q}:=\;J\left(\text{q}\right)\dot{q} \end{array}$$

The redundancy problem lies in the fact that, even with a full-ranked Jacobian matrix, there might exist many (infinite) joint velocities which result in the same velocity at the end-effector **ẋ**. This problem can be solved with a mechanical analogy, imagining that a mechanical manipulator, with negligible inertia and purely viscous (symmetric and positive-definite) joint impedance *W* producing viscous joint torques $W\dot{q}$, is passively moved at the end-effector with an imposed velocity ***ẋ***. This action will produce a *unique joint velocity*

(3) $$\begin{array}{l} \dot{q}=W^{-1}J^{T}\left(\text{q}\right)\underset{B\left(\text{q}\right)}{\underset{︸}{{\left(J\left(\text{q}\right)W^{-1}J^{T}\left(\text{q}\right)\right)}^{-1}}}ẋ \end{array}$$

This type of redundancy solution is also known as a *W*-weighted generalized pseudo-inverse (Klein and Huang, 1983; Doty et al., 1993) but, rather than its derivation, here we want to emphasize its physical interpretation. The highlighted term *B*(***q***): = (*J*(***q***)*W*^−1^*J*^*T*^(***q***))^−1^ represents the *task-space damping*, i.e., the damping force perceived at the end-effector (task-space) while imposing a task-space velocity **ẋ** and solely due to the joint-space damping *W* and its mapping via the Jacobian *J*(***q***).

To accomplish a task such as reaching for a target ***x***_*d*_, a simple way is to “pull” the end-effector toward the target with the action of an *extrinsic spring K*, producing a task-space force ***F*** = *K*(***x***_*d*_−***x***) on the hand always directed toward the target. The first and most basic form of PMP (Mohan and Morasso, 2011) corresponds to the thin-line loop [denoted PMP1 and including *K*, *W*^−1^, *J*(***q***) and *J*^*T*^(***q***)] in Figure 1B. This form of PMP can solve redundancy but is *incompatible* with Donders' law. More details can be found in Tommasino and Campolo (2017) but it is straightforward to see that, once on the target, i.e., ***x*** = ***x***_*d*_, no force is produced by the extrinsic spring (***x*** − ***x***_*d*_ = 0) and any posture ***q*** such that ***x***_*d*_ = *FK*(***q***) will therefore be maintained indefinitely.

To guarantee unique postures, an elastic scalar potential can be introduced in the joint-space, for example, via an *intrinsic stiffness* matrix *K*^*J*^ at the joint-level. This will subject the manipulator to joint torques $\tau_{el}:=\;K^{J}\left(\text{q}-{\text{q}}^{*}\right)$ which continuously drive the manipulator toward a given “rest” posture q^*^ (an equilibrium for the intrinsic elastic potential). The addition of elastic joint torques **τ**_*el*_ leads to the second form of PMP, denoted as PMP2 and highlighted in Figure 1B. As shown in Tommasino and Campolo (2017), this solves redundancy and accounts for Donders's law but does not solve the posture/movement problem due to the contrasting effect of the extrinsic spring *K*, which pulls the end-effector toward the target ***x***_*d*_, and intrinsic stiffness *K*^*J*^ which pulls the whole manipulator toward the “rest” posture q^*^. It is easy to show that, in general, ***x***_*d*_ is not an equilibrium for the system. If ***x*** = ***x***_*d*_, then the extrinsic spring *K* will produce no force (***x***_*d*_ − ***x*** = 0) and the effect of the intrinsic stiffness *K*^*J*^ on the task will not be contrasted, moving the end-effector away from ***x***_*d*_.

The unwanted interference due to intrinsic elastic torques **τ**_*el*_ can be removed by adding the *task-space force*:

(4) $$\begin{array}{l} \lambda_{0}:=\;B\left(\text{q}\right)J\left(\text{q}\right)W^{-1}\tau_{el} \end{array}$$

The task-force **λ**_0_ is the last piece of the puzzle needed to complete the description of the **λ**_0_-PMP model shown in Figure 1B. For a complete theoretical derivation, the reader should refer to Tommasino and Campolo (2017), here we only wish to provide its physical intuition. As mentioned above, **τ**_*el*_ is an elastic torque field responsible for Donders' law, in the sense that it constantly drives the manipulator toward “natural” or “comfortable” postures (Campolo et al., 2011). In doing so, however, it also interferes with the task completion (posture/movement problem). In order to block its effect *only* in the task-space (so, preserving Donders' law in joint-space) one could proceed as follows: the elastic torque **τ**_*el*_, if unblocked, would produce a joint velocity ${\dot{q}}_{el}=W^{-1}\tau_{el}$ with a resulting task velocity ${ẋ}_{el}=J\left(\text{q}\right){\dot{q}}_{el}$. The task-force **λ**_0_ can be seen as the force needed to contrast the (task-space) damping force $B\left(\text{q}\right){ẋ}_{el}=B\left(\text{q}\right)J\left(\text{q}\right)W^{-1}\tau_{el}$. The *novel* concept of a task-space force **λ**_0_ is very useful as it provides a *force perspective* which allows other force-control strategies to be simply *superimposed* onto our postural mechanisms. The extrinsic spring force **F** = *K*(***x***_*d*_ − ***x***) plays the role of *Task Planner*. In fact, as shown in Tommasino and Campolo (2017), other type of force-control strategies could be superimposed, such as a visco-elastic task-space force field or an optimal force field planner minimizing the total task-space force moving the end-effector toward the desired target.

**Remark:** With reference to the diagram in Figure 1B, the task-space damping *B*(***q***) in Equation (3) transforms task-space velocities ***ẋ*** into task-space forces which balance out the effect of the extrinsic spring *K*, leading to the following task-space dynamic equation:

(5) $$\begin{array}{l} B\left(\text{q}\right)ẋ=K\left({\text{x}}_{d}-\text{x}\right) \end{array}$$

Although the task-space damping is posture-dependent and more equations are needed to fully solve the dynamics, some remarkable properties can already be noted: (i) the task-damping *B*(***q***) in Equation (3) directly depends on the intrinsic damping *W* which, therefore, directly affects task-space dynamics (Equation 5); (ii) the intrinsic stiffness *K*^*J*^ does not appear in Equation (5) and therefore does *not* directly affect the task dynamics, however, it does it *indirectly* through postures adjustments in the null-space which affect the Jacobian and therefore *B*(***q***).

Although in this work we only consider elastic joint-space potentials, other potentials (for example due to gravity) and their gradient can be simply added in parallel to the elastic torque **τ**_*el*_ in Figure 1 and, the **λ**_0_ would only block, in task-space, the effects of these posture-dependent torque fields but not velocity-dependent (i.e., dynamic) torques, such as viscous effects due to *W*. The reader is referred to our previous work (Tommasino and Campolo, 2017) for this and other details. The role of the **λ**_0_ also offers a force field perspective to the UCM and LJH hypothesis. By compensating only the task-space components of intrinsic potential fields, the null-space is left *uncontrolled* and motions along task-irrelevant directions are due exclusively to the passive dynamics of the limb under control.

### Application to wrist pointing tasks

Building on previous experimental and computational studies (Campolo et al., 2009, 2010, 2011; Charles and Hogan, 2012; Formica et al., 2012; Tommasino and Campolo, 2017), we are specifically interested in capturing human-like motor strategies during pointing tasks performed with the wrist. To implement the model in Figure 1B, we shall first determine the Jacobian *J* from the forward kinematics; the intrinsic stiffness (*K*^*J*^) matrix and the rest posture q^*^; the damping (*W*) matrix as well as the extrinsic stiffness matrix *K*.

#### Forward kinematics

**Figure 2:**
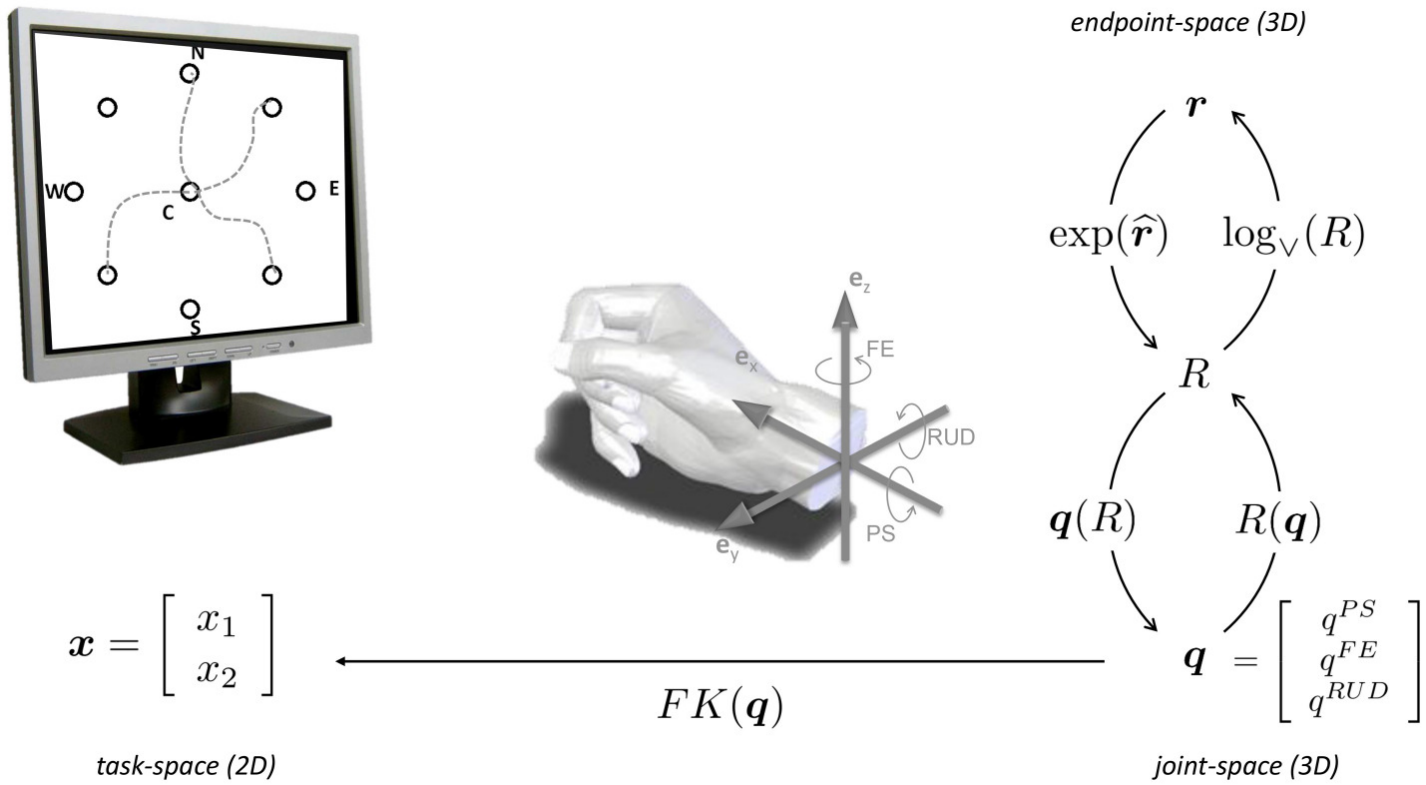
Center-out task and kinematic spaces involved in wrist pointing. 3D wrist configurations can be equivalently expressed in terms of 3 × 3 rotation matrices *R*(***q***), rotation vectors **r** or joint rotations ***q***. The forward kinematics ***x*** = *FK*(***q***) maps 3D wrist orientations onto the 2D task, expressed in screen coordinates ***x***. Adapted from Campolo et al. (2011).

With reference to Figure 2, we assume that the wrist is used to point a virtual laser beam onto a point on a computer screen, i.e., a two-dimensional task-space of coordinates $\text{x}={\left[x_{1}\;x_{2}\right]}^{T}\in {\mathbb{R}}^{2}$. The human wrist is modeled as an ideal, three-dimensional gimbal comprising the following three orthogonal, rotational axes (from proximal to distal):

– the Prono-Supination (PS) axis, aligned along ${\text{e}}_{x}:=\;{\left[1\;0\;0\right]}^{T}$;
– the Flexion-Extension (FE) axis, aligned along ${\text{e}}_{z}:=\;{\left[0\;0\;1\right]}^{T}$;
– the Radial-Ulnar-Deviation (RUD) axis, aligned along ${\text{e}}_{y}:=\;{\left[0\;1\;0\right]}^{T}$.

The joint-space is therefore three-dimensional and can be described via a joint vector ***q*** = [*q^PS^ q^FE^ q^RUD^*]*^T^* ∈ ℝ^3^ or, alternatively, via rotation vectors (Campolo et al., 2010, 2011). as shown in Figure 2.

The forward kinematics (Equation 1) for a 3DOF wrist at distance *d* = 1 m and initially pointing in the [1 0 0]^*T*^ direction can be written as

(6) $$\begin{array}{l} \left[\begin{array}{c} x_{1}\\ x_{2} \end{array}\right]=\underset{\text{screen projection}}{\underset{︸}{\left[\begin{array}{ccc} 0 & -d & 0\\ 0 & 0 & d \end{array}\right]}}\cdot R\left(\text{q}\right)\cdot \left[\begin{array}{c} 1\\ 0\\ 0 \end{array}\right] \end{array}$$

where *R*(***q***) represents the 3D hand orientation, computed as $R\left(\text{q}\right)=\exp\left(-{\hat{\text{e}}}_{x}q^{PS}\right)\exp\left({\hat{\text{e}}}_{z}q^{FE}\right)\exp\left({\hat{\text{e}}}_{y}q^{RUD}\right)$ where the exponential notation $\exp\left(\hat{e}\theta \right)$ represents the rotation about an axis **e** by and angle θ (Murray et al., 1994). Further details are given in Campolo et al. (2011) and references therein. Once the forward kinematics is given, the Jacobian can be analytically computed based on its definition given in Equation (2).

#### Subject-specific intrinsic stiffness *K*^*J*^ and rest posture q^*^ from experimental data

For the 3DOF wrist, the intrinsic stiffness (as well as the damping) is represented by a 3 × 3 *symmetric matrix*. The rest posture q^*^ represents the posture (three joint angles) of minimum elastic energy. Using nonlinear inverse optimization (NIO) techniques (Tommasino and Campolo, 2016), a subject-specific matrix *K*^*J*^ and rest posture q^*^ can be directly derived from experimental data. As detailed in Tommasino and Campolo (2016), experimental data consisting of thousands of data points are fitted to a quadratic surface, typically used in literature to encode Donders' law. This can be seen as an extreme down-sampling of experimental data and the resultant quadratic surface can be seen as an average Donders' surface. The reader is referred to Tommasino and Campolo (2016) for the detailed procedure based on nonlinear inverse optimization. One thing to highlight is that it is the relative *ratio* between eigenvalues of *K*^*J*^ which determines a specific Donders' law, not the absolute values. For this reason, the trace of the matrix can be set to any arbitrary (positive) number. To be in line with biomechanical (passive) stiffness values found in literature (Peaden and Charles, 2014), we set this value to be *trace*(*K*^*J*^) = 4 Nm/rad.

#### Damping *W* and intrinsic time constant

While the intrinsic stiffness matrix is derived directly from a fitting process of experimental data, for the intrinsic damping matrix *W*, rather than trying to use the coefficients of the matrix *W* as “extra degrees of freedom” to better fit experimental data, we assume that *damping is proportional to stiffness*, in line with experimental evidence (Tsuji et al., 1995; Perreault et al., 2004; Tee et al., 2004; Peaden and Charles, 2014). In other words, we hypothesize that the same biomechanical factors which determine the “shape” (in terms of eigenvectors and eigenvalues) of *K*^*J*^ will determine a similar “shape” for *W*. For this reason we set the damping to be proportional to the intrinsic stiffness

(7) $$\begin{array}{l} W=\tau_{0}K^{J} \end{array}$$

where τ_0_ is a scalar (positive) value with the units of time, and can be therefore thought of as an *intrinsic time constant*. The reason is that, for a simple scalar, linear spring-damper system, the ratio between damping and stiffness determines exactly the time constant of the system.

#### Extrinsic stiffness *K* and task-space dynamics

The extrinsic stiffness *K* is responsible for the task-space dynamics together with the task-space damping *B*(***q***), as highlighted in Equation (5). However, *B*(***q***) is determined once *J* and *W* are given, as in Equation (3). In this work, we are considering very simple center-out tasks, as described below. As there is no *a priory* preferential direction in the task-space, we shall consider an *isotropic* extrinsic stiffness *K*, although other choices of task planners are possible (Tommasino and Campolo, 2017) and will be the focus of future works. When the extrinsic stiffness is constant, our computational model predicts trajectories that evolve in time according to a first order dynamic typical of a visco-elastic system with constant parameters. In other words, the desired target is reached with an exponential velocity profiles that decays to zero only after an infinite amount of time. This feature is clearly not bio-inspired as biological movements are characterized by bell-shaped velocity profiles. In the PMP, Morasso and colleagues have overcome this issue with the introduction of a *time base generator*, i.e., a time-dependent gain matrix that rescales end-effector velocities according to a minimum-jerk profiles (Morasso et al., 2010). However, in this work we pursue the approach proposed by Arimoto et al. (2005), as it could reproduce velocity profiles more similar to our experiments. More specifically, a time-varying extrinsic stiffness matrix:

(8) $$\begin{array}{l} K\left(t\right)=k\cdot \left(1-e^{-\frac{t}{\tau }}-\frac{t}{\tau }e^{-\frac{t}{\tau }}\right)\cdot \left[\begin{array}{cc} 1 & 0\\ 0 & 1 \end{array}\right] \end{array}$$

with the property of increasing its stiffness value from zero to *k* with an *extrinsic time constant* τ (notice that when pointing to a new target the time *t* is reset to zero), is used to avoid first order dynamics typical of visco-elastic systems with constant stiffness and damping parameters.

### Example: anisotropic damping and curved task-space trajectories

**Figure 3:**
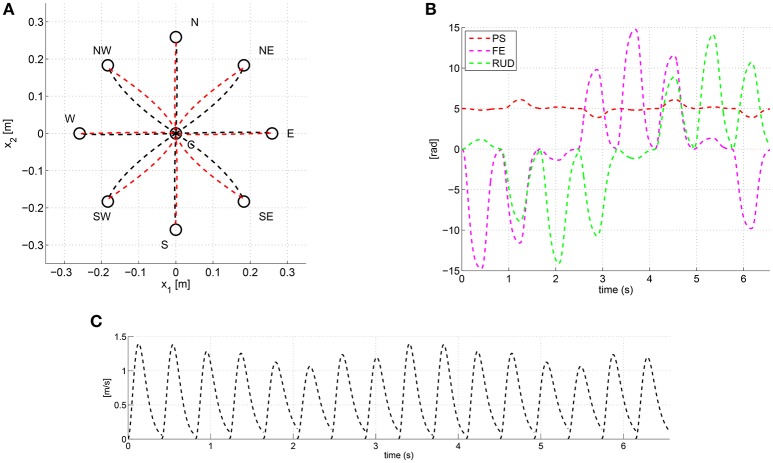
Task space paths **(A)**, joint trajectories **(B)** and task-space tangential velocity profiles **(C)** predicted via the **λ**_0_-PMP with the anisotropic damping *W* of Equation (9).

As an example, Figure 3 shows the trajectories predicted via the **λ**_0_-PMP with an anisotropic intrinsic damping *W*. More specifically, every outbound and inbound movement was simulated for a duration *T* = 0.4 [*s*], and with time-constants τ = τ_0_ = 0.08 [*s*], i.e., one-fifth of *T*. The anisotropic damping *W* was set according to Equation (7) as:

(9) $$\begin{array}{l} W=\tau_{0}K^{J}=0.08\left[\begin{array}{ccc} 0.5 & 0 & 0\\ 0 & 1.5 & 0\\ 0 & 0 & 2 \end{array}\right]\frac{Nms}{rad} \end{array}$$

hence proportional to an anisotropic intrinsic stiffness *K*^*J*^. The rest posture was set as q^*^ = [5 0 0]^*T*^ [*deg*] and the scalar stiffness $k=22.5\left[\frac{Nm}{rad}\right]$.

As shown in Figure 3A the anisotropic damping results into paths of different degree of curvature depending on the specific movement direction. Moreover, outbound and inbound movements follow different paths, especially along the (NW-SE) and (SW-NE) directions. Figure 3B shows the joint space trajectories predicted by the model and Figure 3C shows that task-space tangential velocity profiles are bell-shaped thanks to the time-varying stiffness *K*(*t*) (Arimoto et al., 2005).

## 3. Comparative analysis of task-dynamics: experimental pointing tasks vs. donders-fitted λ_0_-PMP model predictions

In this section, we will compare the *average* experimental task-space trajectories, as previously measured in Campolo et al. (2011) from human subjects during wrist pointing tasks, with those predicted via a *Donders-fitted* **λ**_0_-PMP model, i.e., a **λ**_0_-PMP model for which the *only postural parameters are fitted to capture the Donders' law for a specific subject*. The major limitation of our previous model (Campolo et al., 2011) is that it was limited to static postures, while our current **λ**_0_-PMP model can also generate movements.

The main hypothesis is that a Donders-fitted model **λ**_0_-PMP, i.e., fitted to only capture postural strategies, is also able to display path dynamics such as curved task-space trajectories as experimentally found by Charles and Hogan (2010). A major difference with their experimental paradigm is that their subjects only used FE and RUD movements, as PS movements were restrained, so it was not a redundant task. In our case, subjects are free to rotate the forearm about the PS axis, adding a degree of redundancy.

### Donders-fitted **λ**_0_-PMP model for wrist-pointing tasks

Our **λ**_0_-PMP model in Figure 1B requires specification of a Jacobian [*J*(***q***)], a task-planner (*K*) as well as intrinsic damping (*W*) and intrinsic postural parameters (*K*^*J*^ and q^*^). Once specialized to wrist-pointing tasks and ideally assuming a similar wrist structure for all subjects, the forward kinematics (Equation 6), and therefore the Jacobian *J*(***q***), will be the same for all subjects. On the other hand, we shall fit subject-specific postural parameters *K*^*J*^ and q^*^. Note that these parameters alone only capture Donders' law, i.e., they can identify optimal postures for giving pointing directions (Campolo et al., 2011; Tommasino and Campolo, 2016) but cannot tell where to point. The actual motion, in particular the geometry of task-space trajectories, will be shaped by the task dynamics (Equation 5). Here, rather than fitting every single movement *a posteriori* with a specific damping *W*, we make the *a priori* hypothesis that *intrinsic damping is proportional to intrinsic stiffness*, via an intrinsic time constant as in Equation (7).

As shown in Equation (5), task-space dynamics depend on *K* and on *B*(***q***) which, in turn, depends on the intrinsic damping *W* via Equation (3). For the task-planner, we shall assume an extrinsic *K*(*t*) as in Equation (8) hence isotropic and therefore not directly responsible for path curvatures. Both the intrinsic and extrinsic time constant, in Equations (7), (8) respectively, affect the speed of the simulated trajectory, in particular, the time required for the simulated wrist, to reach the target in “steady-state” (i.e., an equilibrium posture compatible with Donders' law) after the beginning of the movement. In general, movement speed can be target and subject specific, hence we set both time constants to be proportional to the average movement duration *T*_*sj*_ that subject *s* needs to point to the target *j*. More specifically, we heuristically found that, by setting τ = τ_0_ = *T*_*sj*_/5 the model predicts both task-space and joint space dynamics that are compatible with the experimental ones (see results below). Similar to Equation (7), we used the time-constant τ_0_ to tune the scalar stiffness *k* in Equation (8) as:

(10) $$\begin{array}{l} k=b_{max}/\tau_{0} \end{array}$$

where *b*_*max*_ is the maximum eigenvalue of the matrix *B*(***q***_0_) and ***q***_0_ is the initial wrist configuration prior to the starting of the movement. Therefore, *k* was set on a subject-specific (because of *B*(***q***)) and movement-specific basis (because τ depends on the average time *T* that the subject needs to perform the movement).

We know that our model predicts curvatures in task-space, as shown in Figure 3. We shall now compare the average curvature displayed by a specific human subject with the curvature predicted by our **λ**_0_-PMP model, once Donders-fitted to a specific subject.

### Experimental protocol

We asked six subjects to perform center-out pointing tasks toward nine targets on a computer screen. As shown in Figure 2, the nine targets consist of a central target and eight peripheral ones arranged over a circle (with a radius of 15°) and oriented along the eight cardinal directions, i.e., North (N), NorthEast (NE), etc., which also define the naming convention).

Each subject performs 10 trials at self-paced speed. Each trial consists of eight outbound movements (from the center to a peripheral target) and 8 inbound movements (from a peripheral target to the center). In a trial, each peripheral target is visited only once and an outbound movement is always followed by an inbound movement. The order in which targets are visited is computed prior to the start of the trial as a pseudo-random permutation.

Throughout the experiment, subjects grasp a light-weight 3D printed handle mounting an inertial measurement unit (IMU) that records hand orientations *R*(***q***), with respect to the fixed reference frame, at 120 Hz. A computer monitor is used to display the center-out task to the subject. The visual feedback consists of the desired target position (a red circle) and the current location pointed at by the subject (a yellow circle). The current location is displayed at coordinates $\text{x}={\left[x_{1}\;x_{2}\right]}^{T}$ computed as in Equation (1), is updated realtime via Equation (6), where *R*(***q***) is sensed by the IMU.

### Data analysis

In this work, we are mainly interested in task-space dynamics, in particular the fact that trajectories during pointing tasks with the wrist appear more curved than in similar tasks performed with the arm (Charles and Hogan, 2010).

#### Movement start and end times

To this end, we follow the same data analysis method proposed in Charles and Hogan (2010). Specifically, the recorded kinematic data is first filtered with smoothing splines (Dohrmann et al., 1988; Charles and Hogan, 2010) to ease numerical differentiation in estimating task-space velocity profiles. The starting and ending times of a movement are identified from the task-space tangential velocity profiles: the start of movement is set to occur at the time of the first data sample *before* the velocity peak with a value below 20% peak velocity. Similarly, the end of a movement is set to occur at the time of the first data sample *after* the velocity peak with a value below 20% the peak velocity. Movements featuring a path length and/or a duration beyond two interquartile from the (subject-specific) median were excluded from the analysis. To compute the average trajectory of a movement, the data was normalized with respect to the movement time and then linearly interpolated at 20 equally temporally spaced samples.

#### Path curvature and hysteresis

**Figure 4:**
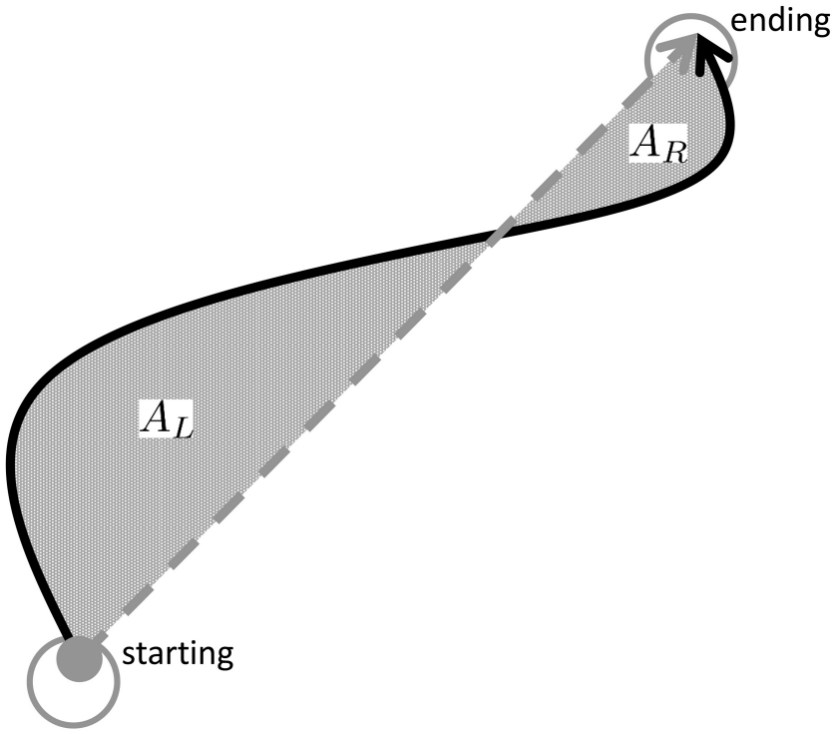
Movement curvature is assessed by calculating the area enclosed to the left (*A*_*L*_) and to the right (*A*_*R*_) of the straight-line connecting the starting and ending of a movement. The total area *A*_*sum*_ = *A*_*L*_ + *A*_*R*_ indicates whether movements are curved and the net area *A*_*net*_ whether there is a tendency to veer more on the right (*A*_*net*_ > 0) or to the left *A*_*net*_ < 0.

To assess path curvature in task-space, we follow the method proposed in Charles and Hogan (2010) to test whether task-space paths are on average curved and if so, whether outbound and inbound movements had different direction of curvatures. More specifically, since each movement occurs between two targets, we consider the straight-line connecting the two targets and directed from the initial target to the final one. This direction is used to determine whether the actual movement is on the left or on the right of the straight line, as depicted in Figure 4. In particular, we shall consider the whole area enclosed between the actual movement and the straight line and split this area into “right” area (*A*_*R*_), i.e., the enclosed area to the right of the straight line and “left” area (*A*_*L*_) as the enclosed area to the left of the straight line. Both left and right areas are defined non-negative and are normalized with respect to the square of the nominal target-to-target distance (${\left(\frac{\pi }{12}\right)}^{2}$ [*m*^2^]). For each movement, either inbound or outbound, we compute the following measures:

– *total area A*_*sum*_: = *A*_*R*_ + *A*_*L*_ (non-negative by definition), indicating deviations of the actual movement from the straight-line.
– *net area A*_*net*_: = *A*_*R*_ − *A*_*L*_, indicating tendency of a path to deviate more on the right (*A*_*net*_ > 0) or to the left (*A*_*net*_ < 0).

Finally, since an outbound movement is always followed by an inbound movement, we also consider the *path hysteresis* defined as $A_{hyst}:=\;A_{net}^{OUT}+A_{net}^{IN}$, i.e., the area enclosed in between outbound and inbound paths.

To assess the statistical significance of each measure, we use a *t*-test (with α = 0.05) to test the following hypotheses: (1) paths are curved (*Asum* ≠ 0); (2) outbound paths have a preferred curvature direction ($A_{net}^{OUT}\neq 0$); (3) inbound paths have a preferred curvature direction ($A_{net}^{IN}\neq 0$); and (4) an outbound-inbound sequence presents hysteresis ($A_{net}^{OUT}\neq -A_{net}^{IN}$).

### Results

For all subjects and for all movements we found *A*_*sum*_ to be statistically different from zero suggesting that task-space paths executed with the wrist are not straight also in presence of redundancy (this was not the case in Charles and Hogan, 2010, where PS was locked).

**Figure 5:**
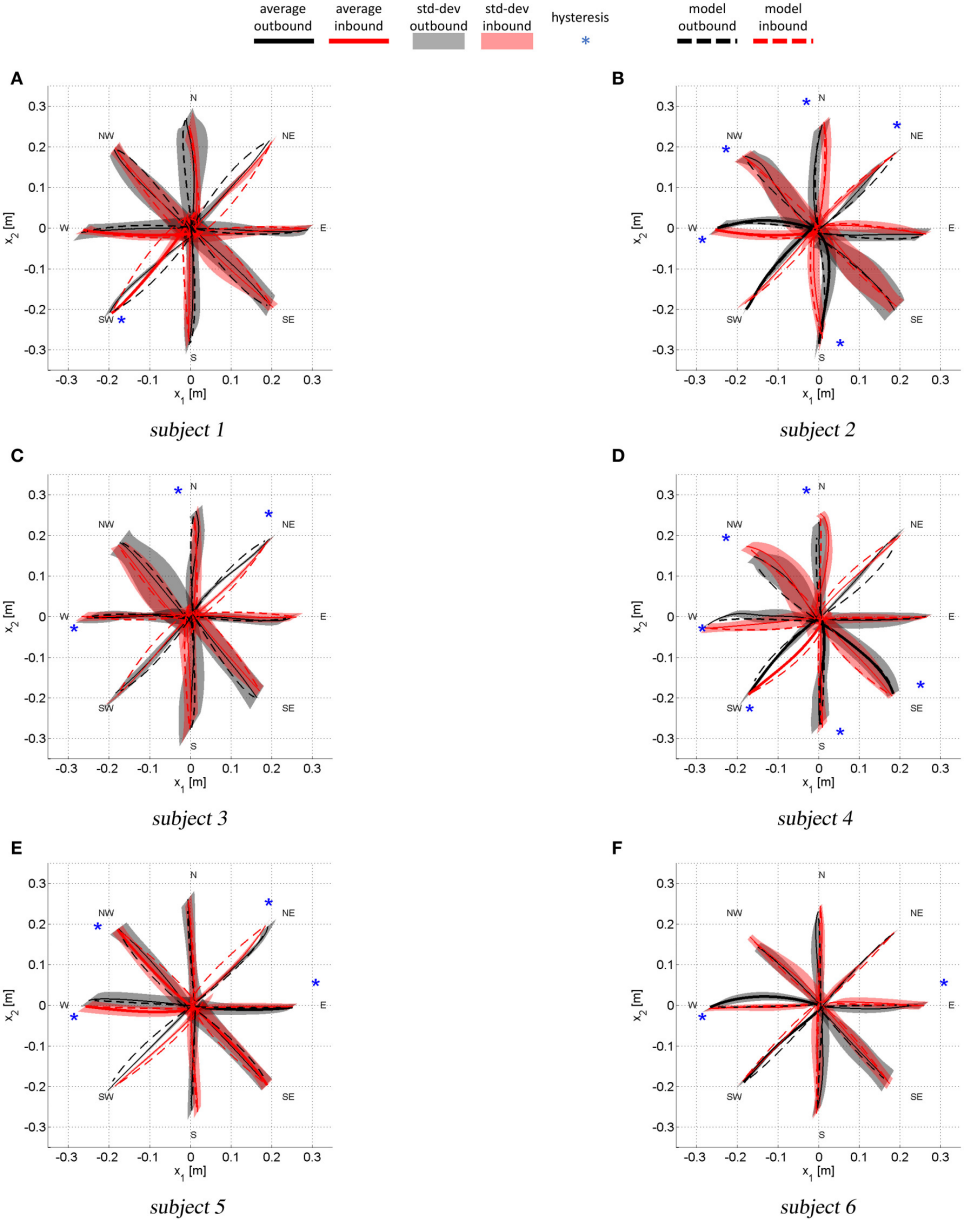
Average task-space trajectories. Shaded areas show the standard deviations for both outbound and inbound movements. Thick lines are relative to paths with *A*_*net*_ statistically different from zero (i.e., a preferred curvature direction). The stars mark movement for which $A_{net}^{IN}$ was statistically different from $A_{net}^{OUT}$, i.e., those movement that present hysteresis.

Figure 5 shows the average outbound and inbound paths of the six subjects together with their standard deviations (shaded areas). Thick lines mark movements for which *A*_*net*_ was statistically different from zero (i.e., a preferred curvature direction) while stars mark segments with statistically significant hysteresis (i.e., outbound and inbound follow different paths). Superimposed with experimental trajectories, Figure 5 also shows the task-space trajectories predicted via a Donders-fitted **λ**_0_-PMP model (dashed lines). The subject-specific postural parameters *K*^*J*^ and q^*^ (estimated with method proposed in Tommasino and Campolo (2016)), used to simulate the model for each subject, are shown in Tables 1–2.

**Table 1:** Subject-specific intrinsic stiffness (*K*^*J*^) parameters estimated via NIO from the average postural strategy.

**$\left[\frac{\text{Nm}}{\text{rad}}\right]$**	**${\text{K}}_{11}^{\text{J}}$**	**${\text{K}}_{12}^{\text{J}}$**	**${\text{K}}_{13}^{\text{J}}$**	**${\text{K}}_{22}^{\text{J}}$**	**${\text{K}}_{23}^{\text{J}}$**	**${\text{K}}_{33}^{\text{J}}$**
Subj 1	1.22	0.07	0.08	1.68	0.03	1.09
Subj 2	1.20	0.04	−0.12	1.47	0.26	1.33
Subj 3	1.25	−0.08	0.00	1.63	0.11	1.12
Subj 4	1.21	0.10	−0.15	1.24	0.29	1.54
Subj 5	1.26	0.00	−0.08	1.21	0.16	1.52
Subj 6	1.30	−0.01	−0.04	1.42	−0.05	1.27

*Stiffness subscript correspond to: 1 = PS, 2 = FE, 3 = RUD, so that the stiffness K_*12*_ corresponds to the stiffness along the PS-FE direction*.

**Table 2:** Subject-specific equilibrium postures (q^*^) estimated via NIO from the average postural strategy.

***[rad]***	**Subj 1**	**Subj 2**	**Subj 3**	**Subj 4**	**Subj 5**	**Subj 6**
$q_{PS}^{*}$	0.26	0.51	0.06	0.36	0.35	0.23
$q_{FE}^{*}$	−0.08	0.11	0.04	0.25	0.12	0.03
$q_{RUD}^{*}$	0.13	−0.11	−0.19	−0.09	−0.07	−0.04

**Figure 6:**
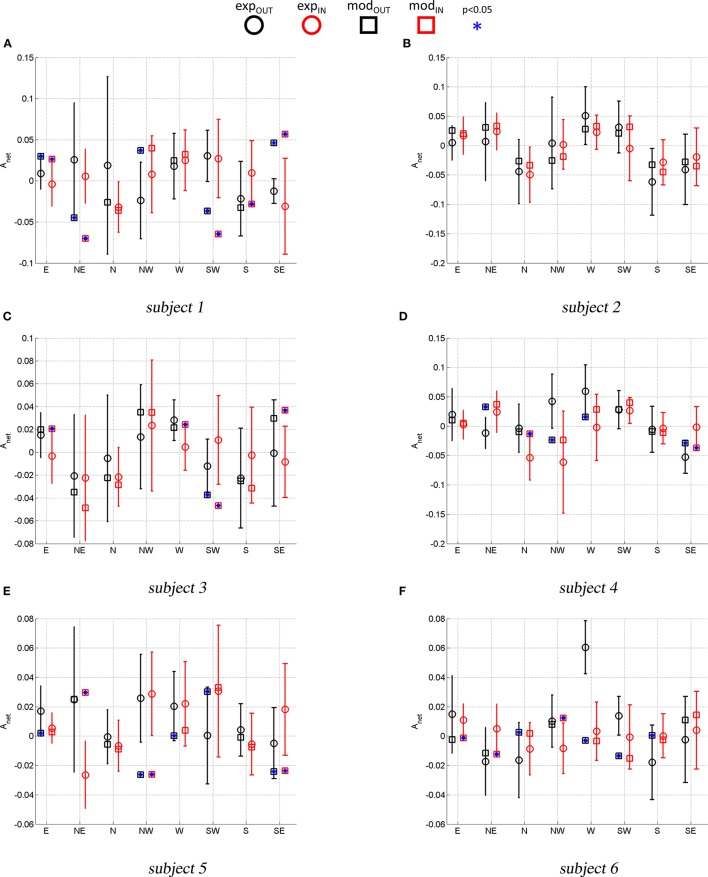
Experimental vs. Model predicted *A*_*net*_. Error bars represent the standard deviation of the experimental ANET. Stars mark a model-predicted Anet that is statistically different (*p* < 0.05) from the average experimental Anet.

Figure 6 compares the experimental *A*_*net*_ (average and standard deviation) with the model predicted *A*_*net*_. This comparison indicates whether the simulated paths have the same curvature direction and magnitude as the experimental ones. A *t*-test (*p* < 0.05) was used for each movement to assess if the average *A*_*net*_ was statistically different from the model predicted *A*_*net*_ (stars).

With reference to Figure 5A, subject 1 shows path hysteresis only for the (SW) target, while, the only statistically different *A*_*net*_ where found for inbound movements from the (W) and the (SW) target (red thick lines) that both veer to the right. With reference to Figure 6A, there is no statistical difference between the model and the experimental curvatures when pointing to and from the (N) and the (W), from (NW) and to (S) targets. The model is particularly accurate in capturing the average curvature of the (N) inbound, the (S) inbound and the (W) outbound and inbound. Overall, for this subject the model can only capture the curvature of 6 out of 16 movement direction (37%).

With reference to Figure 5B, subject 2 presents hysteresis for most of the targets, except for the (E), (SW) and (SE). This subject presents preferred curvature direction when pointing to and from the (W) target, with outbound and inbound both veering to the right. There is also a preference to veer to the right and to the left when performing outbound movements toward the (SW) and (S) targets, respectively. Figure 6B shows that, for all movements, the model predicts curvatures that are not statistically different from the experimental ones.

Similar analysis can be conducted for the remaining subjects. Here we limit ourselves to observe that for subject 3 there were no differences in terms of curvatures in 11 out of 16 movements (about 70% of movements). For subject 4 there were no differences between model and experimental curvatures in 10 out of 16 movements (62% of movements). For subject 5 there were no differences between model and experimental curvatures in 8 out of 16 movements (50% of movements) and for subject 6 in 9 out of 16 movements (56%).

In summary, task-space curvature and hysteresis appear to be subject- and movement-specific and the model can capture most of this features for the majority of movements and subjects.

**Figure 7:**
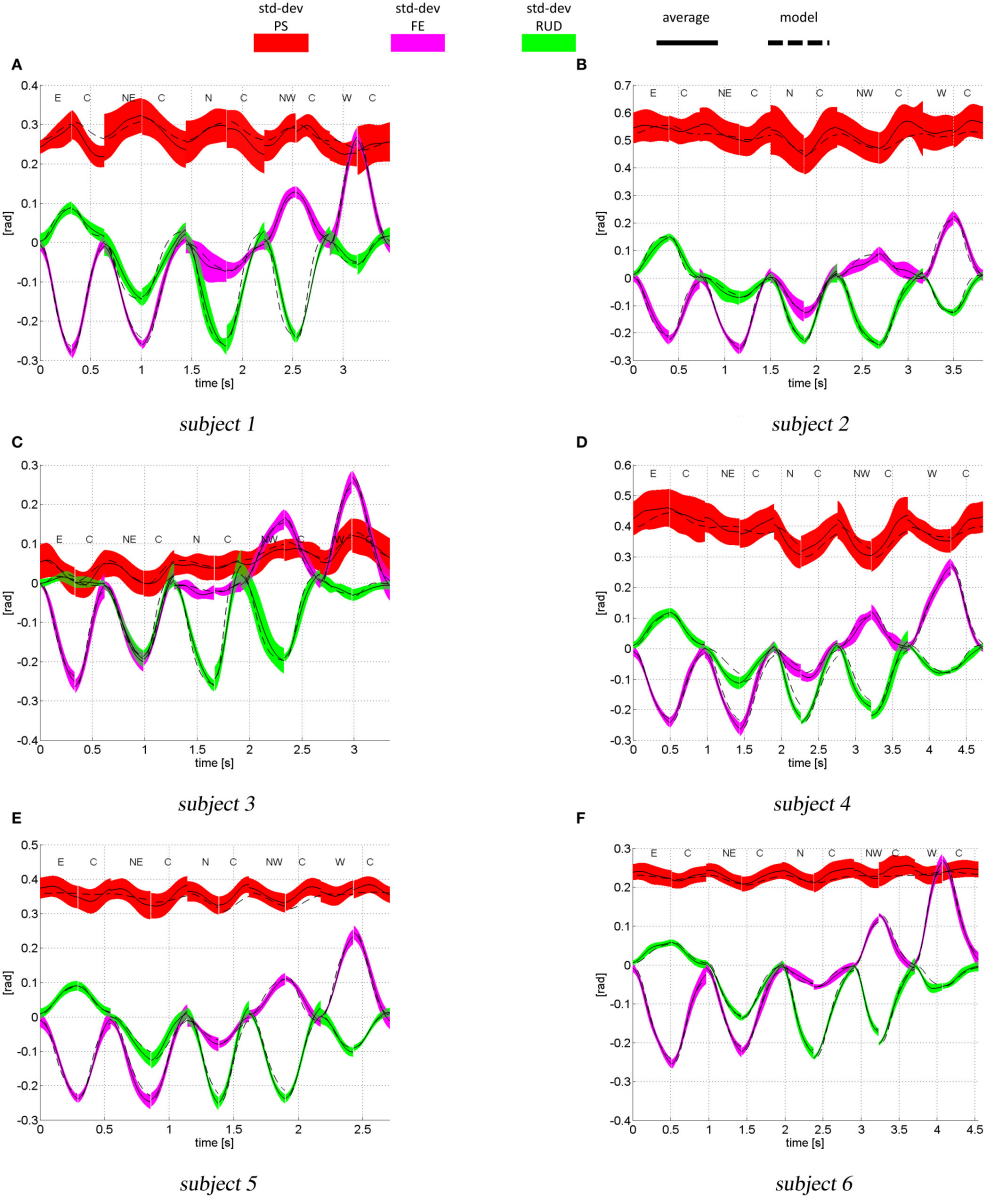
Joint space trajectories (PS in red, FE in magenta and RUD in green), predicted by the model (dashed lines) and measured experimentally: mean (continuous line) and standard deviations (color areas). The letters indicate the target sequence. For instance, the first movement is an outbound movement toward the target (E), the second movement is an inbound movement from target (E) to target (C) and so forth.

The experimental and simulated joint space trajectories are shown in Figure 7 (only outbound and inbound movements from the (E) the (W) target are shown). All subjects show high variability when coordinating the PS rotation (red area), most likely because this is the joint that adds redundancy to the pointing task. The model can accurately reproduce the average FE (magenta color) and RUD (green) trajectories for most of the subjects and movements, while for PS rotations, there are larger errors between the average experimental trajectory and the model.

At the starting and ending times of each movement, i.e., when the wrist is stationary, the model predicted postures only depends on the estimated parameters *K*^*J*^ and q^*^. So, the larger the error between the model and the experimental posture, the less accurate is the estimate of *K*^*J*^ and q^*^. Because we are setting *W* proportional to *K*^*J*^, part of the errors between the model and the experimental trajectories may be due to the error between the real intrinsic subject stiffness and the one estimated from the data. In addition, the model does not take into account inertial and gravitational contributions. While the former has very little effect on wrist and forearm rotations (Peaden and Charles, 2014), gravity torques have been found to be non-negligible (Peaden and Charles, 2014).

**Figure 8:**
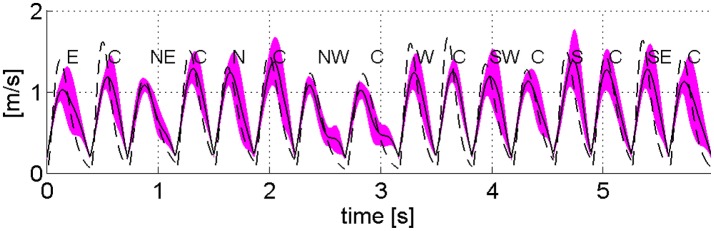
Task space velocity profiles of a representative subject [mean (continuous line) and standard deviation] and those predicted by the **λ**_0_-PMP (dashed line).

Figure 8 compares the experimental task-space tangential velocity profile and those predicted by the model for a representative subject. The time-varying extrinsic spring (Equation 8) reproduces bell-shaped velocity profiles similar to the experimental ones, although, task-space velocities predicted by the model tend to be larger than the experimental ones.

## 4. Conclusion

Motion planning and postural control in the presence of kinematic redundancy continue to be central topics in both neuroscience and robotics. For example, it is still debated why hand movements follows roughly straight-line paths in some experimental conditions while they are curved in others experimental settings. For decades, minimum principles (such as minimum-jerk, minimum variance, and so forth) and optimal control have been used as a tool to model and capture human-like trajectories. Although successful in capturing some features of human movements, when formulated in joint space, these approaches are not only computational demanding but also fail to capture postural control mechanisms such as Donders' law. While it is still unclear how the brain solves redundancy (Mussa-Ivaldi et al., 2011), in robotics kinematic redundancy has been tackled with the task-space control framework that combines local optimization and W-weighted generalized pseudo-inverses. However, as robots start to look more anthropomorphic and to interact with humans, they also need to display natural and intuitive movements and posture. Hence, roboticists are looking at bio-inspired approaches to plan and control task-space trajectories, null-space movements and equilibrium robot postures. We recently addressed the problem of postural control and trajectory planning by combining classical robotic motion planning (velocity resolution control) with neuroscientific evidence and theories of human motor control. We proposed a general and unifying force-field based posture and movement planner that was primarily tested in terms of human-like postural control (equilibrium postural strategies) (Tommasino and Campolo, 2017). In this work we extend our previous results by investigating the trajectory (both in task and joint space) predicted by a specific instance of our general computational framework: the **λ**_0_-PMP. More specifically, we focused on human motor strategies during redundant pointing tasks performed with wrist (and forearm) rotations. In a previous work, Charles and Hogan (2010) showed that when pointing with the wrist, task-space paths are curved and in general, inbound and outbound movements follow different paths. In a successive work, they posited that such features of wrist rotations are due to an anisotropic joint stiffness matrix.

Here, we put forward the hypothesis that anisotropic intrinsic damping, rather than stiffness, is primarily responsible for curved task-space paths. The novel aspect of our approach is that our model was fitted to capture postural strategies and, with the sole hypothesis that intrinsic damping is proportional to stiffness (Equation 7), the model also exhibited curvatures and hysteresis in task-space performance remarkably similar to subject-specific average motions. More specifically, we found that (i) task-space paths are curved also in presence of kinematic redundancy, extending thus the work of Charles and Hogan (2010) where the PS axis was locked; (ii) curvature and hysteresis found in experimental trajectories, on a subject-specific and target-specific basis, are a possible consequence of postural constraints.

It should be noted that our computational framework is capable of generating human-like task-space trajectories from the only knowledge of the terminal target position. Hence, for robotic applications, task-space trajectories must not be pre-programmed as in the classical task-space control approach, but are a direct consequence of the intrinsic and extrinsic impedance parameters (damping and stiffness) used in the model. For the pointing task implemented in this work there is no preferred task-space direction as subjects only receive the final target position as desired target. Therefore, in the model, for the task-space planner we used an isotropic elastic attractor to push the simulated cursor on the desired target. This solution is not only simple but, when combined with suitable intrinsic impedance parameters also results in human-like wrist trajectories. However, as also discussed in Tommasino and Campolo (2017) any task-space force field can in principle be used as task-planner, and therefore, for more complex robotic applications future works will explore the possibility of integrating *dynamic movement primitives* in our framework for the generation of adaptive and compliant skills (Calinon et al., 2013). In summary, in addition to the desired target location, our extended passive motion paradigm requires only the knowledge of: (i) an intrinsic stiffness matrix *K*^*J*^ and an equilibrium posture ***q***_0_ that, combined with the **λ**_0_ force field, allow the prediction of equilibrium (steady-state) wrist postures compatible with experimental (subject-specific) Donders' laws: (ii) the movement duration T, from which both the intrinsic and extrinsic time constants, of the joint-space damping and task-space stiffness respectively, can be set to reach the desired target in T seconds and with an equilibrium posture compatible with Donders' law.

There are of course many approximations and assumptions in our model which, as mentioned, is not meant to predict exact trajectories but rather capturing some basic features of human-like motion. A major limitation is that the intrinsic stiffness *K*^*J*^ is only a very simplified attempt to approximate the real, nonlinear, time-variant mechanical stiffness typically of human arm. This in turn affects not only the predicted postural strategies (i.e., wrist configuration at the beginning and ending of a movement) but also the predicted trajectories as the relationship between damping and stiffness is certainly more complex than the simple proportionality assumed in Equation (7). Furthermore, we only considered an isotropic task planner to investigate the effect of joint damping on path curvatures. However, future works need to compare how different and possibly anisotropic task planners (Tommasino and Campolo, 2017), when combined with anisotropic joint damping, predict subject-specific path's curvature.

A second limitation is that the **λ**_0_-PMP totally neglects feedback, as it is meant to address *motion planning* rather than execution. Our model is however useful at a planning stage, while feedback should be incorporated for movement execution.

As a third limitation, our model is to be considered as a *first order postural and motor planner*, in the sense that it does not take into account the inertial properties of human or robotic arms. This is a specific choice (in some cases an inertia might not even be available, e.g., in motor imagery scenarios) and the model could be extended to include inertial properties. In fact, the role that the manipulator intrinsic inertia would have is the same that the intrinsic damping has in our model. Such an approach would lead to models along the lines proposed by Khatib et al. (2009). Our approach however, is similar to Dietrich et al. (2015) where the manipulator joint stiffness (see Equation 7), compared to manipulator inertia, has been shown to be a more reliable weighting matrix for the calculation of W-weighted pseudoinverse and null-space projector operator.

In conclusion this work presents an extended version of the PMP that can deal with kinematic redundancy in compliance with Donders' law and solve the posture/movement problem. Just like the PMP, our model can find extensive use in planning human-like motions for humanoid robots and, at the same time, be able to capture *natural postures* in compliance with Donders' Law.

## Author contributions

PT and DC wrote and revised the manuscript, and conceived the work; PT performed simulations and data analysis.

### Conflict of interest statement

The authors declare that the research was conducted in the absence of any commercial or financial relationships that could be construed as a potential conflict of interest. The reviewer DH and handling Editor declared their shared affiliation.

## Footnotes

^1^Note that any positive-definite matrix can be used as weight and that in general *W* is configuration-dependent.

^2^The task-space force field is usually generated by combining a desired task-space trajectory with a task-space impedance.
